# Obituary

**Published:** 1874-09

**Authors:** 


### OBITUARY.

Died at Ft. Wayne, Ind., on the 6th of July, Mrs. Dr. S. B. Brown.

It is with feelings of sadness that we chronicle the death of this estimable lady. Mrs. Brown was possessed of those qualities of heart and mind, which could not fail to endear her to a large circle of friends. Of a cheerful social nature, she was a conspicuous and ever welcome member of society in the community where she lived.

She was endowed with superior musical talents, and was always ready with her melodious voice to entertain the social circle or aid in the cause of charity. Only three months before her death she appeared in a principal role in a series of musical entertainments, given in aid of benevolent objects.

The many friends of Dr. Brown in the dental profession, will regard his bereavement with feelings of genuine sorrow and regret.

